# Umbilical Cord-Derived Mesenchymal Stem Cells Ameliorate Nephrocyte Injury and Proteinuria in a Diabetic Nephropathy Rat Model

**DOI:** 10.1155/2020/8035853

**Published:** 2020-04-29

**Authors:** Lian Chen, E. Xiang, Changyong Li, Bing Han, Quan Zhang, Wei Rao, Cuihong Xiao, Dongcheng Wu

**Affiliations:** ^1^Department of Biochemistry and Molecular Biology, Wuhan University School of Basic Medical Sciences, Wuhan, China; ^2^Wuhan Hamilton Biotechnology Co., Ltd., Wuhan, China; ^3^Department of Physiology, Wuhan University School of Basic Medical Sciences, Wuhan, China

## Abstract

Mesenchymal stem cells (MSCs) are shown to alleviate renal injury of diabetic nephropathy (DN) in rats. However, the underlying mechanism of this beneficial effect is not fully understood. The aims of this study are to evaluate effects of umbilical cord-derived mesenchymal stem cells (UC-MSCs) on renal cell apoptosis in streptozotocin- (STZ-) induced diabetic rats and explore the underlying mechanisms. Characteristics of UC-MSCs were identified by flow cytometry and differentiation capability. Six weeks after DN induction by STZ injection in Sprague-Dawley rats, the DN rats received UC-MSCs once a week for consecutive two weeks. DN-related physical and biochemical parameters were measured at 2 weeks after UC-MSC infusion. Renal histological changes were also assessed. Moreover, the apoptosis of renal cells and expression of apoptosis-related proteins were evaluated. Compared with DN rats, rats treated with UC-MSCs showed suppressed increase in 24-hour urinary total protein, urinary albumin to creatinine ratio, serum creatinine, and blood urea nitrogen. UC-MSC treatment ameliorated pathological abnormalities in the kidney of DN rats as evidenced by H&E, PAS, and Masson Trichrome staining. Furthermore, UC-MSC treatment reduced apoptosis of renal cells in DN rats. UC-MSCs promoted expression of antiapoptosis protein Bcl-xl and suppressed expression of high mobility group protein B1 (HMGB1) in the kidney of DN rats. Most importantly, UC-MSCs suppressed upregulation of thioredoxin-interacting protein (TXNIP), downregulation of thioredoxin 1 (TRX1), and activation of apoptosis signal-regulating kinase 1 (ASK1) and P38 MAPK in the kidney of DN rats. Our results suggest that UC-MSCs could alleviate nephrocyte injury and albuminuria of DN rats through their antiapoptotic property. The protective effects of UC-MSCs may be mediated by inhibiting TXNIP upregulation in part.

## 1. Introduction

Diabetic nephropathy (DN) is one of the major microvascular complications of diabetes mellitus (DM) and the leading cause of end-stage renal disease worldwide [1, 2]. About one-third of type 1 DM (T1DM) and a quarter of type 2 DM (T2DM) patients eventually develop DN [3]. DN is clinically characterized by albuminuria and reduced renal function evidenced by a decreased glomerular filtration rate (GFR) and increased serum creatinine and blood urea nitrogen concentration [4]. Morphologically, DN is featured by glomerular mesangial expansion, increased extracellular matrix deposition, thickened glomerular and tubular basement membranes, renal inflammation, and fibrosis [5]. To date, there is no cure for DN. Drugs that control the blood glucose level, decrease blood pressure, or inhibit actions of the renin-angiotensin system may delay but not eliminate the progression of DN [6]. Therefore, development of novel therapeutic strategies that could specifically target the pathogenesis of DN is necessary.

Mesenchymal stem cells (MSCs) are multipotent progenitor cells with ability to self-renew and differentiate into various cell types [7]. MSCs can be isolated from multiple tissues, such as bone marrow (BM-MSCs) [8], adipose tissue (AD-MSCs) [9], and umbilical cord (UC-MSCs) [10]. Evidence accumulated in recent years has revealed that MSCs have multiple biological functions, including immunomodulation, anti-inflammation, antifibrosis, and antiapoptosis properties [11, 12], which offer therapeutic potential for DN. It is known that glomerular podocytes are critical for maintaining the integrity of the glomerular filtration barrier [13] and tubular epithelial cells play an important role in reabsorption of filtered albumin [14]. The development of albuminuria in DN is closely related with apoptosis of glomerular podocytes and tubular epithelial cells, as glomerular vascular lesions lead to increased albumin leakage and tubular damages result in decreased albumin reabsorption. Previous studies have illustrated that transplantation of BM-MSCs or AD-MSCs has therapeutic effects on DN animal models induced by streptozotocin (STZ) [15, 16]. MSCs can protect the kidney from high glucose-mediated histopathological damage and improve renal function of DN, especially improving albuminuria [17, 18]. Compared to BM-MSCs and AD-MSCs, UC-MSCs are younger adult stem cells in origination with stronger multipotency and different paracrine secretomes [19, 20]. Therefore, we hypothesized that UC-MSCs possess potential therapeutic effects on nephrocyte injury and albuminuria through their antiapoptotic property.

In the present study, we have utilized a STZ-induced rat DN model to evaluate the potential protective effects of UC-MSCs on histological and functional injury of DN and to explore whether the therapeutic effect of UC-MSCs is mediated through their antiapoptotic property as well as the underlying molecular mechanisms.

## 2. Materials and Methods

### 2.1. Isolation, Culture, and Characterization of UC-MSCs

Human UC-MSCs were isolated and expanded using a previously described method with modification [21]. Briefly, Wharton's jelly was mechanically dissected into 1-4 mm^3^ pieces and cultured in serum-free complete medium (Lonza, Walkersville, MD). The primary UC-MSCs were expanded by regular subculture and harvested at passage 5 (P5) for experimental usage. The use of UC-MSCs was approved by the Institutional Ethics Review Board of Renmin Hospital of Wuhan University (2019L-G001). Written informed consent was obtained before the umbilical cord collection.

The UC-MSCs were characterized for their phenotype by flow cytometry after staining with antibodies against CD44, CD73, CD90, CD105, CD19, CD34, CD45, and HLA-DR (BioLegend, San Diego, CA) and for their multilineage differentiation potential using osteogenic and adipogenic differentiation media (Weitong Biosciences, Shenzhen, China), respectively, following the manufacturer's instruction.

### 2.2. Animal Experiments

All animal experiments were approved by the Institutional Animal Care and Use Committee of Hubei Provincial Center for Safety Evaluation of Food and Drug (#20200101). Male Sprague-Dawley (SD) rats (*n* = 15) with body weight of 200–230 g were purchased and housed in a specific pathogen-free facility under conditions of 24°C and 12-hour light/dark cycle with diet and water ad libitum. After 1 week of adaptation, diabetes was induced by single intraperitoneal injection of 60 mg/kg streptozotocin (STZ, Sigma-Aldrich, St. Louis, MO) in sodium citrate buffer (0.01 M, pH 4.5) after overnight fasting. Equal volume of sodium citrate buffer was administrated as a vehicle control (control group, *n* = 5). Six weeks after STZ injection, the rats with a blood glucose level over 16.7 mmol/L were recruited and randomly injected with UC-MSCs (2 × 10^6^ cells suspended in 0.5 mL PBS; UC-MSC group, *n* = 5) or 0.5 mL PBS (DN group, *n* = 5) via a tail vein, once a week for consecutive two weeks. The animals were sacrificed at 2 weeks after UC-MSC treatment, and samples of blood and kidney tissues were collected for further analysis.

### 2.3. Physical and Biochemical Analysis

Body weight and blood glucose level of rats were monitored during the experiment. Serum and urine creatinine concentration were measured using a creatinine assay kit (Jiancheng Bio, Nanjing, China). Blood urea nitrogen (BUN) concentration was measured using a BUN assay kit (Jiancheng Bio, Nanjing, China). Urinary albumin concentration was measured with an enhanced BCA protein assay kit (Beyotime, Shanghai, China).

### 2.4. Histological Examination

The kidneys of rats were removed, fixed in 4% paraformaldehyde, embedded in paraffin, and cut into 5 *μ*m sections. The sections were deparaffinized, hydrated, and stained with hematoxylin and eosin (H&E), Periodic Acid-Schiff (PAS), and Masson Trichrome, respectively. Renal injury and morphological disorders were observed under a microscope (Olympus, Tokyo, Japan). Semiquantitative scoring was performed to assess extent and intensity of extracellular matrix deposition and fibrosis in the glomeruli and tubulointerstitium using an arbitrary unit: 0, normal; 1, mild; 2, moderate; and 3, severe [22].

A TUNEL assay was carried out using the In Situ Cell Death Detection Kit (Roche, Shanghai, China) according to the manufacturer's instructions. Briefly, renal tissue sections were treated with 0.1% Triton X-100 (Servicebio, Wuhan, China) for 20 min at room temperature, blocked with 3% H_2_O_2_, and then placed in working solution (10% enzyme solution and 90% label solution) for 1 hour at 37°C. The apoptotic cells were stained dark brown by adding diaminobenzidine (DAB; Servicebio, Wuhan, China) and observed under a microscope (Olympus, Tokyo, Japan).

### 2.5. Western Blot Analysis

Protein was extracted from homogenized renal tissues in lysis buffer containing a cocktail of proteinase inhibitors (Beyotime, Shanghai, China). Protein concentrations were determined using a BCA protein assay kit (Beyotime, Shanghai, China). Proteins (50 *μ*g) were separated by sodium dodecyl sulfate-polyacrylamide gel electrophoresis (SDS-PAGE) and transferred onto PVDF membranes (Millipore, Bedford, MA). After blocking with 5% nonfat milk, the membranes were incubated with indicated primary antibodies against Bax, Bcl-xl, thioredoxin 1 (TRX1), thioredoxin-interacting protein (TXNIP), phospho-P38 MAPK, P38 MAPK (all 1 : 1000 dilution; CST, Danvers, MA), phospho-apoptosis signal-regulating kinase 1 (p-ASK1), or ASK1 (both 1 : 500 dilution; Affinity Biosciences, Changzhou, China) at 4°C overnight. Subsequently, the membranes were incubated with horseradish peroxidase- (HRP-) conjugated secondary antibody (1 : 5000; Bio-Rad, California, USA) for 60 min. The protein bands were visualized using enhanced chemiluminescence (ECL; Bio-Rad, California, USA) reagents and captured with the ChemiDoc system. Intensity of the protein bands was semiquantified and normalized against GAPDH using ImageJ analysis software.

### 2.6. Enzyme-Linked Immunosorbent Assay (ELISA)

The concentration of high mobility group protein B1 (HMGB1) in kidney tissue was determined using a rat HMGB1 ELISA kit (Mlbio, Shanghai, China) according to the manufacturer's instructions. The absorbance at 450 nm was measured using a model ST-360 microplate reader (Kehua Bio, Shanghai, China). All measurements were performed in triplicate.

### 2.7. Statistical Analysis

Statistical analysis was performed using GraphPad Prism 5 software (GraphPad Software Inc., California, USA). Results were expressed as the mean ± standard error of mean (SEM). Data were analyzed by a *t*-test, and *p* < 0.05 was considered statistically significant.

## 3. Results

### 3.1. Characterization of UC-MSCs

**Figure 1:**
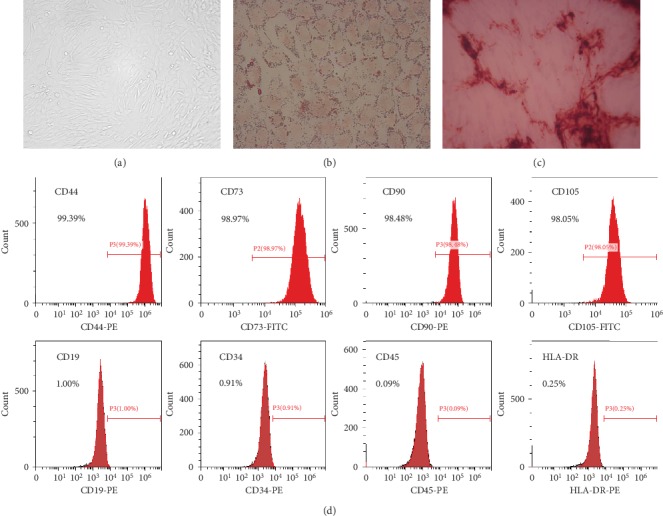
Characterization of UC-MSCs: (a) plastic adherence and fibroblast-like morphology of UC-MSCs (magnification ×200); (b) adipogenic differentiation of UC-MSCs (Oil Red O staining, magnification ×400); (c) osteogenic differentiation of UC-MSCs (Alizarin Red staining, magnification ×100); (d) UC-MSCs express cell surface markers such as CD44, CD73, CD90, and CD105 but do not express CD19, CD34, CD45, and HLA-DR.

The *in vitro* expanded UC-MSCs at passage 5 (P5) used in this study were characterized by plastic adherence and their typical fibroblast-like morphology (Figure 1(a)), multilineage differentiation potential (Figures 1(b) and 1(c)), and immunophenotype (Figure 1(d)).

### 3.2. UC-MSCs Improve Renal Function of DN Rats

**Figure 2:**
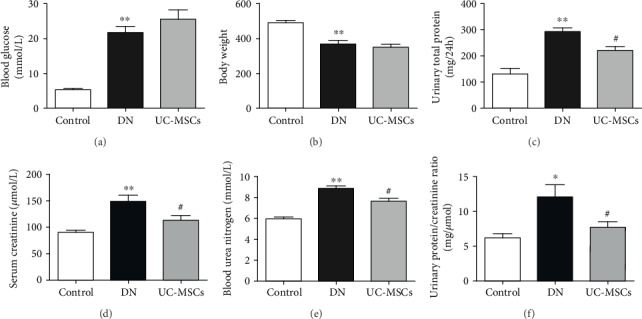
Physical and biochemical analysis of rats. The diabetic model was established by increased blood glucose (a) and reduced body weight (b). Renal function of rats was assessed by 24-hour urinary total protein (c), serum creatinine (d), blood urea nitrogen (e), and urinary protein/creatinine ratio (f) (*n* = 4~5, ^∗^*p* < 0.05 versus control group, ^∗∗^*p* < 0.01 versus control group, and ^#^*p* < 0.05 versus DN group).

The establishment of STZ-induced diabetes in the rats was determined by the significant increase of blood glucose (21.82 ± 1.76 mmol/L versus 5.38 ± 0.40 mmol/L) with loss of body weight (367.66 ± 22.96 g versus 492.92 ± 12.69 g) (Figures 2(a) and 2(b), *p* < 0.01). The diabetic nephropathy (DN) of STZ-treated rats was demonstrated by the significant increase of 24-hour urinary total protein (292.17 ± 15.58 mg/24 h versus 131.43 ± 21.26 mg/24 h), serum creatinine (148.82 ± 12.22 *μ*mol/L versus 91.13 ± 3.45 *μ*mol/L), blood urea nitrogen (8.84 ± 0.27 mmol/L versus 5.96 ± 0.18 mmol/L), and urinary protein/creatinine ratio (12.13 ± 1.77 mg/*μ*mol versus 6.22 ± 0.62 mg/*μ*mol) (Figures 2(c)–2(f), *p* < 0.01) in comparison with the control group. Compared with the DN group, UC-MSC treatment decreased 24-hour urinary total protein (222.11 ± 14.72 mg/24 h), serum creatinine (113.77 ± 8.46 *μ*mol/L), blood urea nitrogen (7.67 ± 0.28 mmol/L), and urinary protein/creatinine ratio (7.74 ± 0.79 mg/*μ*mol) (Figures 2(c)–2(f), *p* < 0.05). These data indicate protective effect of UC-MSCs on renal function of DN rats.

### 3.3. UC-MSCs Alleviate Histological Injury in Kidney of DN Rats

**Figure 3:**
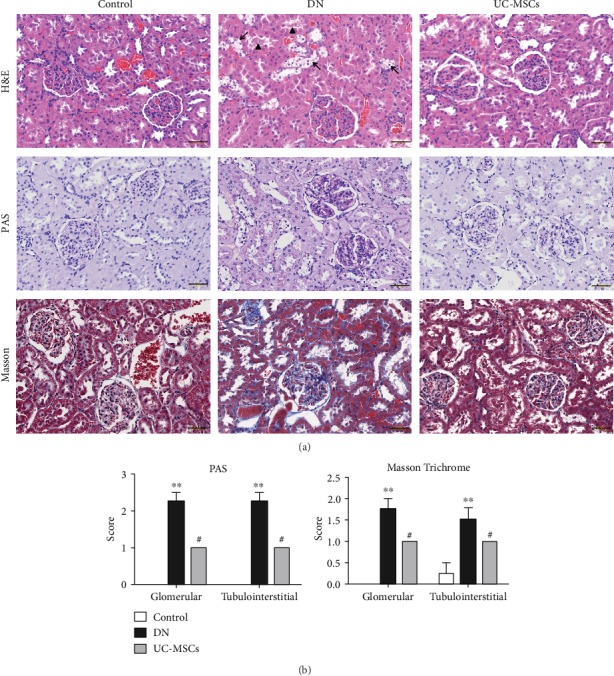
Histological examinations of renal tissues were conducted in rats. Pathological changes of kidney were evaluated by H&E, PAS, and Masson Trichrome staining (a), scale bar = 50 *μ*m (black arrows indicate vacuolation of tubular epithelial cells, and arrowheads indicate cylinder in H&E staining). Semiquantitative analysis of PAS and Masson Trichrome staining was conducted as previously described (b) (*n* = 4~5, ^∗∗^*p* < 0.01 versus control group, ^#^*p* < 0.05 versus DN group).

Histological examinations of kidney were performed using H&E, PAS, and Masson Trichrome staining. H&E staining showed glomerular hypertrophy, vacuolation of tubular epithelial cells, and cylinder in DN rats (Figure 3(a), upper row). PAS staining showed enhanced extracellular matrix deposition inside glomeruli and on the basement membrane of the tubule (Figure 3(a), middle row). Significant glomerular and tubulointerstitial fibrosis was also detected in the kidney of DN rats by Masson Trichrome staining (Figure 3(a), lower row). In the UC-MSC-treated group, the pathological abnormalities above were remarkably alleviated as shown in Figure 3(a) (right column). The pathological damages in the kidney of DN rats and the beneficial effects of UC-MSC treatment were further confirmed by semiquantitative scoring on renal tissues stained with PAS and Masson Trichrome, respectively (Figure 3(b)). These histological results were consistent with renal function changes mentioned above, and both together implicate strong therapeutic potential of UC-MSCs on diabetic nephropathy.

### 3.4. UC-MSCs Reduce Renal Cell Apoptosis by Inhibiting TXNIP Upregulation

**Figure 4:**
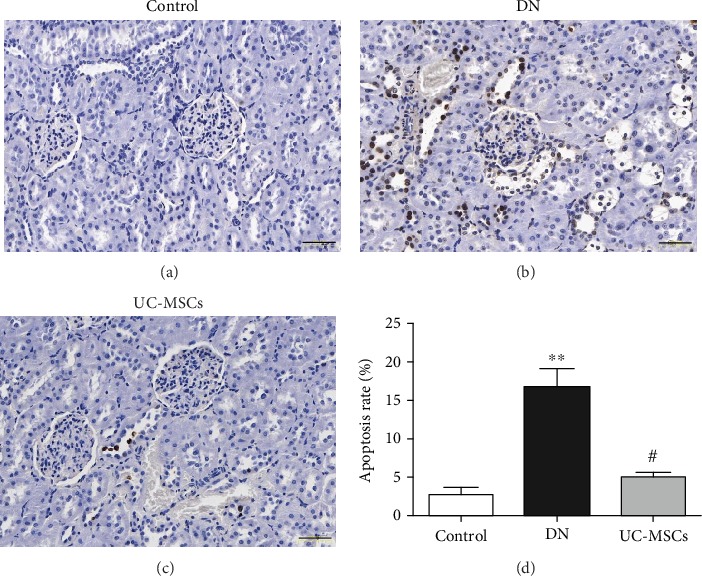
Renal cell apoptosis was detected by the TUNEL assay (a–c) and quantified as the percentage of apoptotic cells (d), scale bar = 50 *μ*m (*n* = 5, ^∗∗^*p* < 0.01 versus control group, ^#^*p* < 0.05 versus DN group).

The TUNEL assay was performed (Figures 4(a)–4(c)) to determine whether the beneficial effects of UC-MSCs on renal injuries in DN rats are associated with their antiapoptotic property. There was a significant increase of apoptotic cells (stained in brown) in the DN group compared with the control group (Figure 4(d), *p* < 0.01), which was decreased by UC-MSC treatment (Figure 4(d), *p* < 0.05).

**Figure 5:**
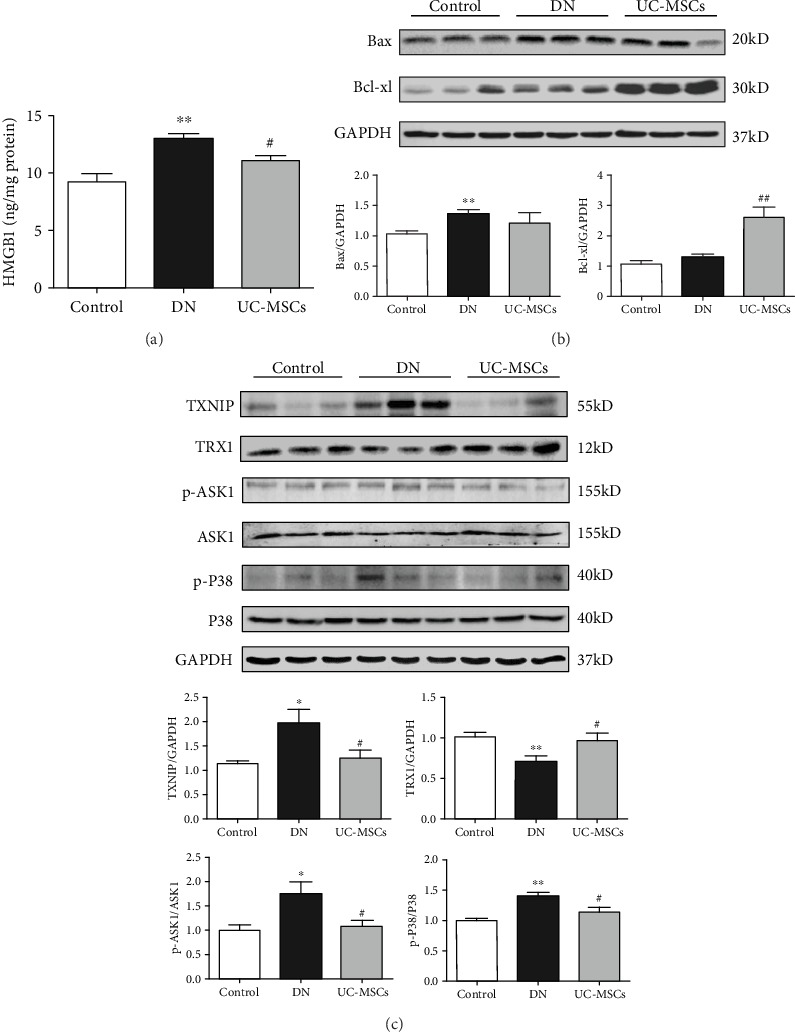
Apoptosis-related proteins were examined in the kidney of rats. (a) Concentration of HMGB1 in renal tissue was determined by ELISA. (b, c) Expression of Bax, Bcl-xl, TXNIP, TRX1, p-ASK1, ASK1, p-P38, and P38 was detected by the Western blot assay. GAPDH was used as the internal control (*n* = 4~5, ^∗∗^*p* < 0.01 versus control group, ^∗^*p* < 0.05 versus control group, ^##^*p* < 0.01 versus DN group, and ^#^*p* < 0.05 versus DN group).

Several apoptosis-related markers were examined to explore the potential molecular mechanisms of the antiapoptotic effect of UC-MSCs in detail. The ELISA result showed that expression of high mobility group protein B1 (HMGB1) in the kidney of DN rats (12.96 ± 0.46 ng/mg protein) was significantly higher than that in control rats (9.21 ± 0.73 ng/mg protein) (Figure 5(a), *p* < 0.01), which was downregulated by UC-MSC treatment (11.08 ± 0.42 ng/mg protein) (Figure 5(a), *p* < 0.05). The Western blot assay was conducted to further examine the apoptosis-related proteins expressed in renal tissues. The results showed that expression of Bax and thioredoxin-interacting protein (TXNIP), both proapoptotic proteins, was upregulated in the DN group compared with the control group (Figure 5(b), *p* < 0.01; Figure 5(c), *p* < 0.05), while the expression of Bcl-xl and thioredoxin 1 (TRX1), both antiapoptotic proteins, was significantly upregulated in the UC-MSC group compared with the DN group (Figure 5(b), *p* < 0.01; Figure 5(c), *p* < 0.05). Moreover, the phosphorylated level of apoptosis signal regulating kinase 1 (ASK1) and P38 was clearly enhanced in the DN group compared with the control group (Figure 5(c), *p* < 0.01), which was downregulated after UC-MSC treatment (Figure 5(c), *p* < 0.05). Considering the essential role of TXNIP playing in stress-induced cell apoptosis, these results indicate that the antiapoptotic effect of UC-MSCs on renal cells is probably mediated by inhibiting TXNIP upregulation.

## 4. Discussion

In the present study, we investigated the protective effects of UC-MSC transplantation on STZ-induced renal injury in DN rats, and we further explored the possible mechanisms. We found that UC-MSCs decreased 24-hour urinary total protein, serum creatinine, blood urea nitrogen, and urinary protein/creatinine ratio in DN rats. Morphologically, UC-MSCs attenuated glomerular hypertrophy, vacuolation of tubular epithelial cells, and cylinder in DN rats. UC-MSCs also reduced extracellular matrix deposition inside glomeruli and alleviated glomerular and tubulointerstitial fibrosis in DN rats. Taken together, UC-MSCs improved renal function and histological damage in the kidney of DN rats. Furthermore, UC-MSC treatment reduced the apoptosis rate of renal cells in DN rats.

Multiple groups have studied the underlying mechanisms of MSC antiapoptotic effects in various organ injury models. Two main mechanisms have been proposed. (1) MSCs secrete various growth factors, such as IGF1, VEGF, and HGF [23–25]. (2) There is increased expression of proregenerative/antiapoptotic genes and/or possibly mRNA transfer to injured cells by MSCs or MSC-derived microvesicles or exosomes [15, 26]. High mobility group protein B1 (HMGB1) is a member of damage-associated molecular patterns (DAMPs), which is usually released from damaged or dead cells during apoptosis [27]. In the present study, the ELISA result showed that the expression of HMGB1 was increased in the kidney of DN rats and decreased after UC-MSC treatment, which suggests that UC-MSCs have protective effect on renal cells of DN rats. The Western blot assay showed upregulation of antiapoptotic protein Bcl-xl in UC-MSC-treated rats, which also demonstrates that UC-MSCs have antiapoptotic effect on renal cells.

Apoptosis signal-regulating kinase 1 (ASK1), a redox-regulated apoptosis signal kinase, is usually bound to thioredoxin 1 (TRX1) under basal conditions [28]. TRX1 is a small redox protein that controls reactive oxygen species (ROS) levels and limits cell apoptosis from oxidative stress, while thioredoxin-interacting protein (TXNIP) could inhibit the antioxidant function of TRX [29]. TXNIP is a nucleoprotein which could be significantly upregulated by hyperglycemia [30]. Once TXNIP shuttles to the cytosol from the nucleus under high glucose condition, TXNIP binds to TRX1 and the ASK1-TRX1 complex is disrupted. The TXNIP-TRX1 complex could inhibit TRX1 in response to excessive ROS, which results in oxidative stress and cell apoptosis [31]. As a redox-sensitive protein, HMGB1 translocates from the nucleus to the cytosol under oxidative stress stimulation, which results in further damages to renal cells [32]. Meanwhile, TXNIP binds to TRX1 and inhibits its ability to bind ASK1 thereby activating ASK1. The phosphorylated ASK1 continues to activate P38 MAPK and P38 MAPK-mediated apoptosis reaction [33]. In the present study, we observed upregulation of TXNIP, downregulation of TRX1, and phosphorylation of ASK1 and P38 MAPK in DN rats, which eventually results in renal cell apoptosis and impaired renal function. However, UC-MSCs downregulated the expression of TXNIP induced by hyperglycemia and reduced the following oxidative stress and apoptosis reaction in renal cells, which significantly alleviated nephrocyte injury and improved renal function of DN rats.

## 5. Conclusions

We have demonstrated that UC-MSCs attenuate histological and functional injury in the kidney of DN rats. UC-MSCs alleviate nephrocyte injury and proteinuria of DN rats through their antiapoptotic property. The antiapoptotic effect of UC-MSCs may be mediated by inhibiting TXNIP upregulation to some extent. Therefore, transplantation of UC-MSCs may be a new strategy for the treatment of DN, and TXNIP may be a new target for the treatment of DN.

## Data Availability

The datasets used to support the findings of this study are available from the corresponding author upon request.
